# EGFR inhibitors switch keratinocytes from a proliferative to a differentiative phenotype affecting epidermal development and barrier function

**DOI:** 10.1186/s12885-020-07685-5

**Published:** 2021-01-05

**Authors:** Nicolas Joly-Tonetti, Thomas Ondet, Mario Monshouwer, Georgios N. Stamatas

**Affiliations:** 1 Johnson & Johnson Santé Beauté France, 1 Rue Camille Desmoulins, 92787 Issy-les-Moulineaux, France; 2 Janssen Pharmaceutical Research and Development, Discovery Sciences, Turnhoutseweg 30, 2340 Beerse, Belgium

**Keywords:** Cutaneous adverse drug reactions, Oncology therapy, Tyrosine kinase inhibitors, Skin barrier impairment, Keratinocyte differentiation, Epidermal growth factors receptor inhibitors

## Abstract

**Background:**

Cutaneous adverse drug reactions (CADR) associated with oncology therapy involve 45–100% of patients receiving kinase inhibitors. Such adverse reactions may include skin inflammation, infection, pruritus and dryness, symptoms that can significantly affect the patient’s quality of life. To prevent severe skin damages dose adjustment or drug discontinuation is often required, interfering with the prescribed oncology treatment protocol. This is particularly the case of Epidermal Growth Factor Receptor inhibitors (EGFRi) targeting carcinomas. Since the EGFR pathway is pivotal for epidermal keratinocytes, it is reasonable to hypothesize that EGFRi also affect these cells and therefore interfere with the epidermal structure formation and skin barrier function.

**Methods:**

To test this hypothesis, the effects of EGFRi and Vascular Endothelial Growth Factor Receptor inhibitors (VEGFRi) at therapeutically relevant concentrations (3, 10, 30, 100 nM) were assessed on proliferation and differentiation markers of human keratinocytes in a novel 3D micro-epidermis tissue culture model.

**Results:**

EGFRi directly affect basal keratinocyte growth, leading to tissue size reduction and switching keratinocytes from a proliferative to a differentiative phenotype, as evidenced by decreased Ki67 staining and increased filaggrin, desmoglein-1 and involucrin expression compared to control. These effects lead to skin barrier impairment, which can be observed in a reconstructed human epidermis model showing a decrease in trans-epidermal water loss rates. On the other hand, pan-kinase inhibitors mainly targeting VEGFR barely affect keratinocyte differentiation and rather promote a proliferative phenotype.

**Conclusions:**

This study contributes to the mechanistic understanding of the clinically observed CADR during therapy with EGFRi. These in vitro results suggest a specific mode of action of EGFRi by directly affecting keratinocyte growth and barrier function.

## Background

The epidermis consists of a stratified epithelium, mainly composed of keratinocytes. It provides the first defense of the host against external aggressors including pathogens and prevents dehydration by controlling the rate of transcutaneous water loss. This barrier is highly depended on the keratinocyte differentiation processes, from basal layer cells to terminal corneocytes in the *stratum corneum*. Oncology treatments target proliferative cells primarily using kinase inhibitors. Since the epidermal epithelium normally includes proliferative cells, it is reasonable to hypothesize that it also becomes a target of such therapies [1], a process that can lead to Cutaneous Adverse Drug Reactions (CADR) as consequence of defective epidermal differentiation, alteration of skin equilibrium and barrier dysfunction [2].

Tyrosine kinase inhibitors (TKi) target members of various growth factor receptors, such as the receptors of the Epidermal Growth Factor (EGF), the Vascular Endothelial Growth Factor (VEGF) and the Platelet-Derived Growth Factor (PDGF) as well as the Human EGF Receptor 2 (HER2). Over-activation of these pathways in tumors leads to increased cell proliferation, angiogenesis and genetic abnormalities and suppression of apoptosis [3, 4]. Patients who initially respond to the TKi will generate resistance due to mutations within the 9 to 13 months following the initiation of their therapy, requiring a switch of the therapeutic regiment to address the appearance of such mutations [5, 6]. The first generation of TKi developed in the early 2000s, was followed by the development of the second and third generation of drugs to thwart the appearance of mutations in tumor cells. The third generation of EGFRi irreversibly inhibits EGFR despite the appearance of T790M mutation improving progression-free survival and reduction of CADR compared to standard chemotherapies [7, 8].

Chronic TKi treatments may also directly affect proliferative keratinocytes at the basal level of the epidermis, reducing cell growth rates, cell migration and promoting cell apoptosis, cell attachment, keratinocyte differentiation and pro-inflammatory cytokine expression [9, 10]. In this case, the resulting epidermal structure disturbance and skin barrier dysfunction could contribute to the clinically observed skin rash, pruritus, xerosis, hand-foot skin reaction, nail and hair alterations. Such CADR, also associated with pain and secondary infections, appear in 45–100% of patients receiving TKi and can significantly affect the patients’ quality of life [5]. Medical examination by both dermatologists and oncologists to understand the nature and severity of the symptoms and the body surface area that is affected is necessary to prevent progression to more severe symptoms. Dose adjustment or even drug administration discontinuation could be required, leading to a disturbance of the oncology treatment protocol [11]. Paradoxically however, in some cases the appearance of skin rashes during treatment is correlated with better survival of the patient [12].

To assess the effects of kinase inhibitors on the epidermis, cultures of keratinocytes were exposed to such therapy molecules during 3 days in high calcium conditions to induce keratinocyte differentiation and generate a 3D-stratified differentiated epidermis. Drug impact on the epidermal development was assessed via various markers such as:

- Ki-67, an universally expressed protein among proliferating cells and absent in the quiescent cells [13].
- Filaggrin, a filament-associated protein that binds to keratin fibers and is a marker of terminal differentiation [14].
- Desmoglein-1, a component of desmosomes and differentiation marker expressed in all epidermal layer above the basal layer [15].
- Involucrin, an early differentiation marker expressed in the spinous and granular layers and a protein precursor of the epidermal cornified envelope layer [16].

In order to better understand the emergence of CADRs, we specifically examined in this work the effects of TKi on the epidermal physiology and the eventual consequences on the quality of the skin barrier against external irritant and pathogen penetration and water loss.

## Methods

### Determination of unbound plasma drug concentration

The drug concentrations for the in vitro experiments in this work were selected in a range relevant to therapeutic concentrations (3–100 nm), compared to higher concentrations in the micromolar to millimolar range previously used in literature [17, 18]. These concentrations appeared to be more relevant to study the long-term effect of treatments on the epidermis. Using published research, we identified the maximal drug concentration in plasma following a single daily-recommended dose (Table 1) of drug in a healthy patient. We also identified the percentage of unbound fraction to plasma protein or calculated it using C_max_ plasma concentration and the percentage of unbound fraction in the plasma. Plasma protein binding of the TKi from the study ranged from 0.3 to 5%. The highest unbound plasma concentration was for erlotinib (80 nM) [26] and the lowest for dacomitinib (0.42 nM) [29]. EGFRi equilibrium dissociation constants (K_D_) have been reported by Klaeger et al. [24]. It was possible to compare the EGFRi used in this study. EGFRi drug potencies, ranging from afatinib with a K_D_ of 2 nM and erlotinib, a first-generation drug, with a K_D_ of 2164 nM, (Table 1). Surprisingly, the potency of osimertinib, a third generation of EGFRi, was not decreased compared to another second-generation drug such as the afatinib, lapatinib and dacomitinib. VEGFRi K_D_ were compared using literature.

**Table 1:**
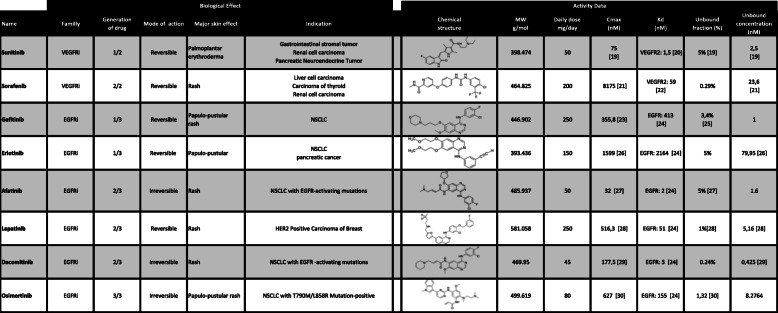
Biological and activity data of VEGFRi and EGFRi. Biological effect data were compiled from the information provided by the web site drugcentral.org (accessed in November 2019). The “major skin effect” presented here is the most frequent cutaneous adverse reaction reported by the FDA Adverse Event Reporting System. NSCLC: non-small cell lung cancer. Drug K_D_ and the determination of plasma concentration after a single dose administration in human are reported here from literature. Data from Klaeger et al. 2017 were used to compare drug K_D_ for EGFRi. Unbound plasma drug fraction was determined as a concentration at nanomolar scale in the literature

### Drug preparation

The selected drugs corresponding to plasma relevant concentrations (3, 10, 30, 100 nM) following administration of a single dose and 1 μM drug concentration were prepared from a 10 mM stock solution dissolved in DMSO. Consequently, the final DMSO concentration was 0.01% for the highest concentration 1 μM. Vehicles were composed of the same DMSO volume as the drug treatment. Acetaminophen was used as a negative control in the same proportion of DMSO.

### Cell culture

Keratinocytes were isolated from donated human tissue after obtaining permission for their use in research applications by informed consent or legal authorization. All cell lines were recently tested for mycoplasma contamination.

### Assessment of skin barrier function

The effect of drugs on the skin barrier function was assessed by measuring trans-epidermal water loss (TEWL) rates on SkinEthic™ Reconstructed Human Epidermis (RHE) model (Episkin, Lyon France) using Tewitro® TW 24 (Courage+Khazaka electronic GmbH, Köln, Germany). This instrument allows 24 simultaneous measurements on RHE. TEWL was analyzed at 33 °C in an incubator. Measurement was performed after 1 h TEWL stabilization and 5 min average of TEWL measurement was performed. Experiments were performed in triplicate and results were normalized to 100% to the TEWL of the control. SDS 0.5% in PBS was added at the surface of the RHE to damage the epidermis structure and consequently increase the TEWL (positive control). Petrolatum, a highly hydrophobic hydrocarbon, water-repelling and insoluble in water was used to block water evaporation at the RHE surface (negative control). Afatinib was added at 100 nM in the media and renewed every 2 or 3 days to simulate chronic drug exposure. DMSO was used in the same proportion in the vehicle.

### EpiScreen™ protocol

The following model was developed by CYTOO (Grenoble, France). Human epidermal keratinocytes cells (HPEKs) from a juvenile Caucasian donor (CellnTec, Switzerland) were grown in a defined proprietary medium from CEllnTech (CnT-PR-D Bern, Switzerland). Keratinocytes were seeded at passage 6 into EpiScreen plates containing collagen 1 coated disc micropatterns (CYTOO, France). Four hours later, unattached cells were washed off and a high calcium medium was added to induce keratinocyte differentiation. The day after, keratinocytes were treated with screening compounds, and Trichostatin A was added at 0.3 μM as an internal positive control. After 3days of treatment, micro-epidermises were fixed with a 10% formalin solution for 30 min, then permeabilized with 0.1% Triton. Several immunostainings were performed: actin (Acti-Stain 555, PHDH1, Cytoskeleton), nuclei (Hoechst, H3570, Invitrogen), and one biomarker of interest per well either anti-involucrin (HPA055211, Sigma), anti-filaggrin (HPA030189, Sigma) or anti-desmoglein-1 (HPA022128, Sigma). Antibodies were added overnight at 4 °C before staining with secondary antibody, anti-rabbit 488 (711–545-152, Jackson) for 2 h at room temperature.

### Images acquisition and analysis

Images of each well were acquired with the Operetta HCS platform (Perkin Elmer) using a × 10 objective in confocal mode in eight z-planes from 2 μm to 44 μm in steps of 6 μm in each of the 3 channels: actin, nuclei, and one biomarker of interest. The first step of the image analysis consisted of detecting micro-epidermis structures on the first z-plane by segmenting the actin staining. Micro-epidermis structures were validated based on several area and roundness min and max criteria. Then, the area of each biomarker staining was measured inside the valid micro-epidermis masks through each z-plane. For all homogeneous biomarker staining, their intensity through the different planes was measured.

### Reconstruction of 3D micro-epidermis images

Based on 50 to 80 micro-epidermis structures per well, an “average” 3D image was created to represent the micro-epidermis phenotype in this well. Micro-epidermis structures were detected using the actin staining in the first z-plane and selected based on area and roundness criteria.

The actin network of each micro-epidermis was analyzed in each z-plane in order to determine the average 3D structure edges. The biomarker intensity was measured in each z-plane for each structure, and then averaged with the other results generated in the same well. Based on the data generated in the two previous steps, an average 3D reconstruction image was generated. It consisted of a meshwork that delimits the structure edges, and a color scaled volume that corresponded to the biomarkers distribution and expression.

### Viability and proliferation assay

The Water-Soluble Tetrazolium Salts (WST-8) Colorimetric Cell Proliferation Kit (Promokine, Heidelberg, Germany) provides a rapid and sensitive way to quantify proliferation and cell viability. Cell proliferation causes an increase in the amount of formazan dye formed that can be quantified by measuring the absorbance of the dye solution at 440 nm using a microtiter plate reader (Perkin Elmer EnVision 2103 Multilabel Reader, Waltham, MA, USA). Keratinocytes from healthy donors were grown in Epilife (ThermoFisher, Waltham, MA, USA) to induce an increase in the activity of mitochondrial dehydrogenases, which cleave the tetrazolium salt WST-1 into formazan. 15,000 Keratinocytes per well were seeded on 96-well plate once confluence was attained the concentration that was+/− ½ log of the plasmatic concentration was added. Ten microliter of Colorimetric Cell Viability Kit was added and completed with 360 μl of culture media, results were read after 4 h at 450 nm to determine cell viability. Results were obtained from 6 donors of keratinocytes in 2 experiments.

### Caspase-3 Fluorometric assay kit

The kit (biotium, Fremont, CA, USA) provides a homogenous assay system for fast and highly sensitive detection of caspase-3 activity by fluorescence in enzymatic reaction or mammalian cells. The fluorogenic substrate (Ac-DEVD)2-R110 contains two DEVD (Asp-Glu-Val-Asp) tetrapeptides and is completely hydrolyzed by the enzyme in two successive steps. Cleavage of the first DEVD peptide results in the monopeptide Ac-DEVD-R110 intermediate, which has absorption and emission wavelengths similar to those of R110 (Ex/Em = 496/520 nm) but has only about 10% of the fluorescence of the latter. Hydrolysis of the second DEVD peptide releases the dye R110, leading to a substantial fluorescence increase. Keratinocytes from healthy donors were grown in Epilife (ThermoFisher, Waltham, Ma, USA) were plated at 15,000 cells per well in 100 μl of medium in a 96-well black microplate. They were allowed to attach and grow overnight in a 37 °C, 5% CO2 incubator. They were then treated for 20 h with a 1:2 dilution series of staurosporine, a caspase 3 inducer [31]. Imaging was performed on Perkin Elmer EnVision 2103 Multilabel Reader using an excitation wavelength of 490 nm and emission wavelength of 535 nm. Cells were incubated at room temperature for 15 min, protected from light. Results were obtained with 6 donors of keratinocytes in 2 experiments.

### Statistics

Results are expressed as means +/− SD. All experiments were performed at least in triplicate. Statistical analysis was performed using one-way analysis of variance (ANOVA) and Student’s t-test. Statistical significance for the difference between the two groups was accepted at the level of *p* < 0.05.

## Results

The effect of 8 oncology molecules, selected from first generation EGFRi and pan-kinase inhibitors, which mostly target VEGFR, and second and third generation therapies targeting main mutations relating to first generation treatment resistance, were assessed in vitro using a 3D micro-epidermis model. The drug incubation concentrations (3, 10, 30, 100 nM) were selected to reflect the clinically relevant (unbound) drug exposures (Table 1). The drug impact was assessed by analysis of tissue size and keratinocyte proliferation using Ki-67 staining and keratinocyte differentiation using filaggrin, desmoglein-1 and involucrin staining.

By covering a large spectrum of concentrations (3 nM to 1 μM), we showed significant changes in the studied parameters, often in a dose-dependent manner. Using such a concentration range allows us to overcome nonspecific interactions of the drugs (e.g. with the plastic support, the extracellular matrix, the proteins in media, etc.) that might interfere in the experiment.

Increased micro-epidermis size and Ki-67 staining with a concomitant decreased of filaggrin, desmoglein-1 and involucrin expression were considered as a pro-proliferation effect of the tested molecule. On the other hand, a pro-differentiation effect was defined as a decrease of both the micro-epidermis size and Ki-67 staining and an increase of filaggrin, desmoglein-1 and involucrin expression.

### Pan-kinase inhibitors barely impact the micro-epidermis structure and differentiation markers

Sunitinib had no impact on the epidermis size and sorafenib strongly decreased the size of the epidermis (Table 2). Both pan-kinase inhibitors did not impact the desmoglein-1 and involucrin protein expression and significantly decreased filaggrin protein expression. Of note, this effect of pan-kinase inhibitors was achieved at 100 nM, lower concentrations did not impact the markers followed in the study. Sunitinib was the only TKi assessed that did not shown any toxicity at the highest concentration tested (1 μM). These results indicate that VEGFRi have a pro-proliferation effect on the keratinocytes.

**Table 2:**
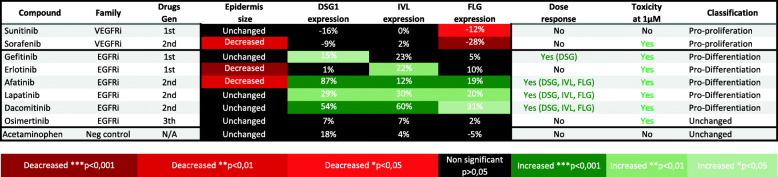
Micro-epidermis physiology is impaired following exposure to EGFRi and VEGFRi. Size of the epidermis and the expression of the protein junction desmoglein-1 (DSG1), involucrin (IVL) and filaggrin (FLG) was assessed by immunostaining and compared to untreated control. Drugs were classified by family and drug generation. Dose responses were determined by comparison of the variation of protein expression at 3, 10 and 30 nM for EGFRi and 3, 10, 30 and 100 nM for the VEGFRi. * *p* < 0.05 at 30 nM (EFGRi) or 100 nM (VEFGRi); ** *p* < 0.01 at 30 nM (EFGRi) or 100 nM (VEFGRi) and *** *p* < 0.001 at 30 nM (EFGRi) or 100 nM (VEFGRi)

### EGFRi affect epidermal structure and differentiation markers

Most of the EGFRi tested, including afatinib, lapatinib, and dacomitinib, showed an effect on desmoglein-1, involucrin and filaggrin expression in a dose-dependent manner (Table 2). Gefitinib increased in a dose-dependent manner only the expression of desmoglein-1. Erlotinib and osimertinib did not affect the expression of the junction proteins. For all EGFRi tested, the epidermal toxicity evaluated at 1 μM was significant, interfering with the epidermal development, to the point that no tissue was available for further data analysis. At unbound plasma drug concentrations 3, 10, and 30 nM, all first and second generation EGFRi showed a decrease in keratinocyte proliferation, micro-epidermis size and an increase of the desmoglein-1, involucrin and filaggrin protein expression, evidence of a pro-differentiation effect.

Interestingly, the osimertinib, a third generation of EGFRi developed to target drug resistance cells but also to provide better drug tolerance, was the only EGFRi which did not show any impact on all parameters except cell toxicity at the higher concentration (1 μM).

### Afatinib affects keratinocyte protein expression, viability and proliferation

**Fig. 1:**
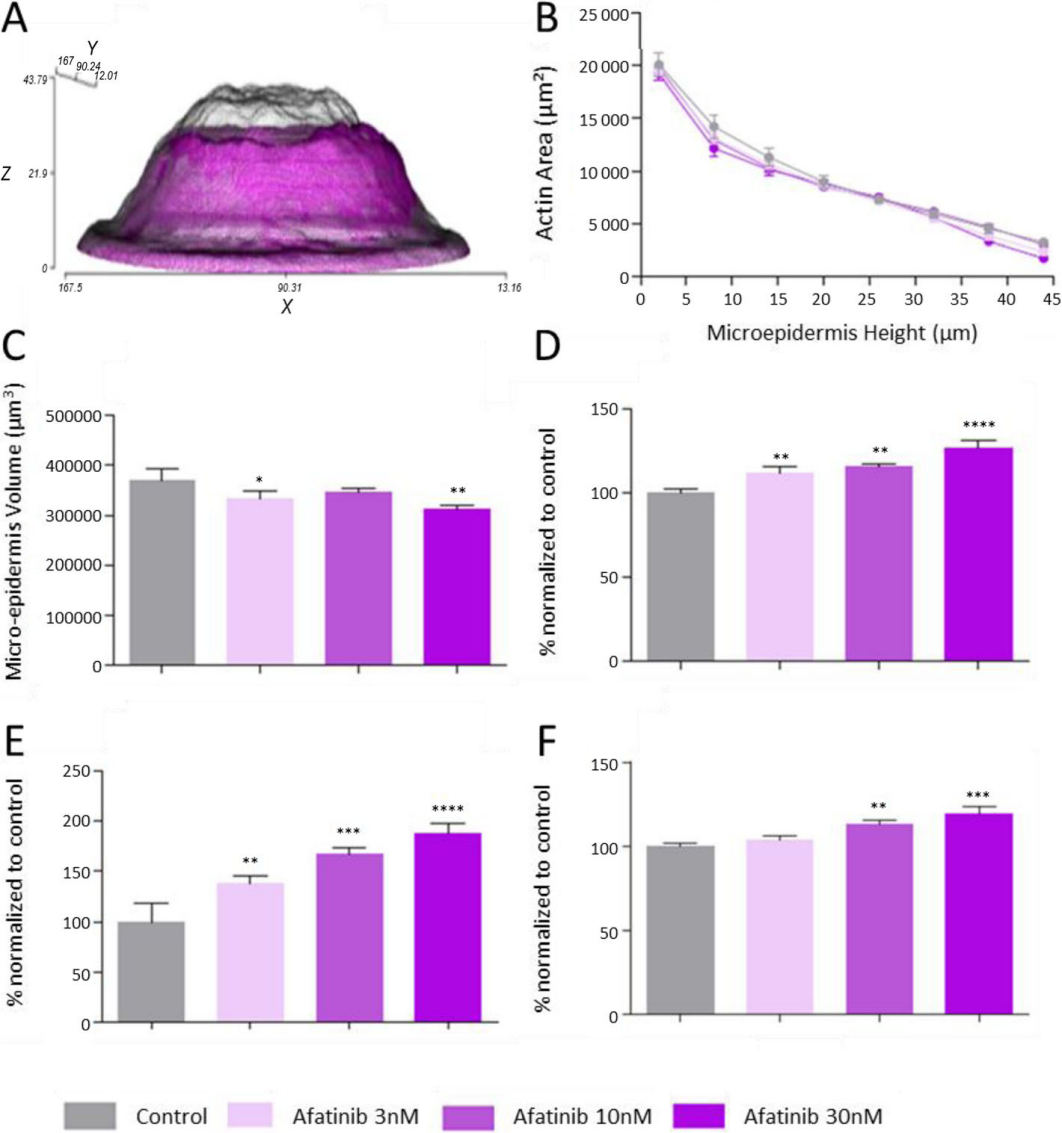
Afatinib decreases the size of the epidermis and increases skin differentiation markers. Micro-epidermises were treated with afatinib at 3, 10 and 30 nM. Drugs and concentrations effect on microepidermis were assessed with different parameters (**a**) Micro-epidermis volume incubated with afatinib 30 nM. **b** actin expression intensity, **c** microepidermis volume, **d** desmoglein-1 expression, **e** Involucrin expression and **f** filaggrin expression. * *p* < 0.05, ** *p* < 0.01, *** *p* < 0.001

Afatinib treatment resulted in significantly decreased epidermal volume in the 3D reconstructed micro-epidermis model compared to vehicle (Fig. 1a). Involucrin and desmoglein-1 expression were significantly increased at 3, 10, 30 nM in a dose-dependent manner and filaggrin expression was significantly increased at 10 nM and 30 nM in a dose-dependent manner. A higher drug concentration above 1 μM was toxic leading to epidermal necrosis.

**Fig. 2:**
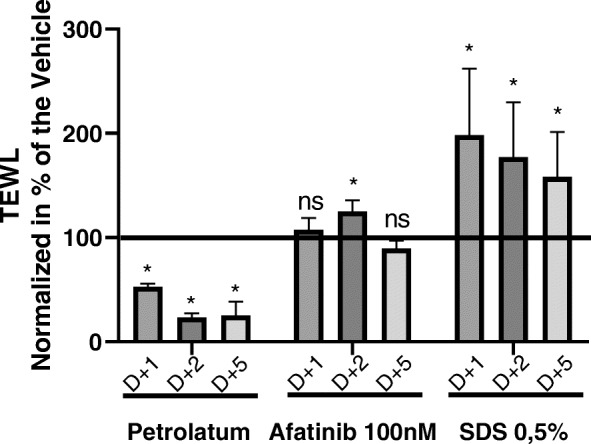
RHE skin barrier function is deteriorating on day 2 of afatinib treatment. Skin barrier function was assessed by measuring the rate of trans epidermal water loss. Topical application of petrolatum on the RHE was used as negative control and topical exposure to a 0.5% SDS solution on the RHE was used as positive control

The effect of Afatinib on the epidermal barrier function was assessed on RHE models by measuring the rate of TEWL (Fig. 2). Addition of petrolatum (negative control) led to a significant decrease of the TEWL rate by 48, 77 and 75% respectively on day 1, 2 and 5 following application, compared to untreated control. The surfactant Sodium Dodecyl Sulfoxide (SDS, at 0.5% used as positive control) significantly increased the TEWL rate by 98 77 and 58% respectively on days 1, 2, and 5 following application. Afatinib significantly increased the rate of TEWL by 22% on day 2, while on days 5 and 7 no significant change was observed.

**Fig. 3:**
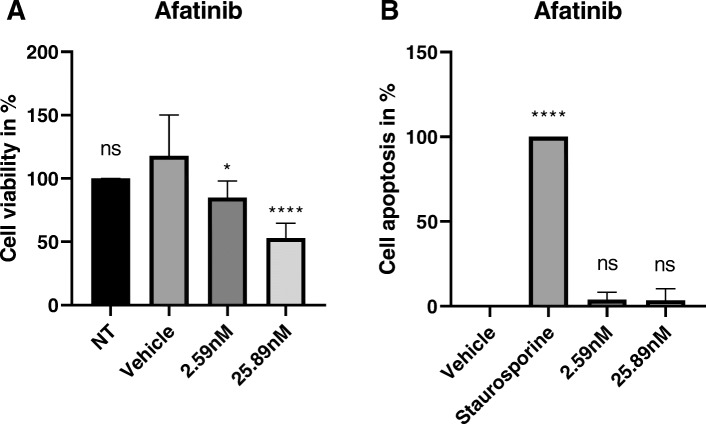
**a** Keratinocyte viability decreases following exposure to afatinib. **b** Afatinib does not induce apoptosis in keratinocytes. Keratinocytes were exposed for 24 h to each condition shown. Percentages represent the relative effect compared to vehicle. Staurosporine at 1 μM was used as positive control and correspond to 100% of cell apoptosis. Post-hoc Dunett’s test * *p* < 0.05, **** *p* < 0.0001

Further results show that afatinib had a significant effect on cell viability in a dose-dependent manner (Fig. 3). On the other hand, afatinib did not show any effect at 2.59 nM and 25.89 nM on cell apoptosis. Taken together these results show that afatinib impairs keratinocyte viability and proliferation in the micro-epidermis model, but it does not induce keratinocyte apoptosis.

Acetaminophen used as control showed no effect on any of the measured parameters including cell toxicity at 1 μM.

## Discussion

The emergence of the TKi in the treatment of cancer has successfully increased the five-year patient survival rate. EGFRi and VEGFRi have led to considerable progress in the treatment of various solid tumors since their introduction and the new generation has considerably increased their efficiency [23]. By targeting proliferative cells, oncology treatments can provoke CADR that potentially disrupt the treatment protocol and impact the patient quality of life [7]. The mechanisms leading to CADR are still poorly understood. Thus far, the effects of TKi on keratinocytes are still unknown and published research has dealt only with relatively high drug concentrations without considering the relevant plasma concentration affecting keratinocytes in a chronic manner. The results presented in this work provide for the first time a better understanding of the mode of action of oncology treatment on the pathophysiology of CADR.

Despite the growing rate of immunotherapy, TKi are extensively used in oncology [33]. Compared to immunotherapies, TKi may impact the epidermal homeostasis differently. In this article, we focused on different generations of TKI to bring a better understanding of their adverse reaction during therapy.

For a better comprehension of the drug impact on the epidermal function, we assessed the direct effects of TKi on epidermal physiology and the consequences on the skin barrier structure based on keratinocytes separate from effects of the immune system. Although, the immune system plays an important role in the appearance and maintenance of CADR, we did not consider the aggravating factor of its dysregulation on epidermal homeostasis.

Sunitinib and sorafenib, two Pan kinase inhibitors mainly targeting VEGFRi have unbound plasmatic fractions of 2 nM and 23.6 nM respectively with equilibrium dissociation constants (K_D_) of 1.5 nM and 59 nM. The study on micro-epidermis performed in the same concentration range had no effect on the epidermal structure, only filaggrin expression was significantly increased for both Sunitinib and Sorafenib. Of note, sunitinib was the only drug in our panel that did not lead to keratinocyte toxicity at 1 μM. It is conceivable that the negative effects of VEGFRi on skin may potentially arise from an impairment of the skin vascularization disturbing keratinocyte growth [34]. The absence of effect using sorafenib at the higher concentration could confirm our hypothesis.

In contrast, EGFRi clearly affect keratinocyte growth at the basal layer leading to a decrease of the epidermal volume in the micro-epidermis model. Afatinib leads to a decreased epidermal volume at 3 nM. On the other hand, afatinib increased the expression of desmoglein-1, involucrin and filaggrin, indicating that EGFRi promote late terminal differentiation while decreasing keratinocyte proliferation at the basal layer.

Osimertinib had surprisingly no impact on epidermal physiology in our model. These data taken together with an high K_D_ value (155 nM) (Table 1) compared to previous drug generations can be explained by the fact that osimertinib targets main mutations identified in EGFR (T790 and p.C797S). These mutations arose after selection pressure of prolonged treatment with first and second generation drugs [35]. However, osimertinib was designed with a higher affinity against the mutated form compared to another drug generation. The wild type (WT) form is targeted but with a lower affinity [36] and consequently barely affects the WT EGFR form of keratinocytes.

Afatinib is an irreversible inhibitor of EGFRi associated with the lowest unbound plasma concentration, C_max_ and K_D_ of the panel. Afatinib impacts all parameters of the study (i.e. epidermis size, skin barrier markers). Consequently, we focused more specifically on the dose response of afatinib (Fig. 1) at 3, 10 and 30 nM corresponding to the range of the unbound plasma concentration to determine keratinocyte growth in the epidermal development. The micro-epidermis size was significantly reduced at 3 and 30 nM. Moreover, involucrin, desmoglein-1 and filaggrin were increased in a dose-dependent manner. Taken together, afatinib affects all markers studied by decreasing keratinocyte proliferation at the basal layer and inducing keratinocyte differentiation, an effect that has a measurable impact on skin barrier function.

In summary, as an early response to drug exposure the skin barrier function is disturbed, as evidenced by the increased TEWL rates on day 2 (Fig. 2). For longer times of drug exposure, keratinocytes undergo a switch from a proliferative to a differentiative phenotype by increasing the expression of structural epidermal proteins including filaggrin, desmoglein-1 and involucrin, as observed on day 3 of the micro-epidermis model. These results would also explain the restoration of TEWL rates by day 5. The transitional period before arriving into a new steady state could directly impact the basal proliferative keratinocytes, able to renew the epidermis, and could explain the appearance of rashes and dry skin that become clinically evident a few weeks following drug exposure [2]. We can hypothesize that drug exposure quickly impacts keratinocyte homeostasis leading to an increase of epidermal permeability that reaches a maximum on day 2. Upon first drug exposure, keratinocytes enhance their differentiation process leading to an increased junction protein expression that could explain the improved epidermal permeability that is observed after day 3.

Clinically CADR symptoms have been reported to appear within the first days of the treatment and then disappear, only to reappear one to 2 months after continuous exposure to oncology drugs [37]. Our results indicate that afatinib induces an early increase in TEWL, which is in agreement with clinical observations. Late manifestation of CADRs may relate to the decreased proliferation and cellular fatigue.

Further analysis was performed to elucidate the effect of afatinib on keratinocytes. The apoptotic activity of keratinocytes was not affected at either dose tested indicating that size reduction of the epidermis is not related to apoptosis, but it is rather linked to a decreased cell number (Fig. 2)

All these data suggest that CADRs are provoked by a decreased keratinocyte proliferation impairing skin regeneration and leading to epidermal size reduction, rather than by inducing keratinocyte apoptosis in the epidermis.

Finally, the new generation of oncology treatment using immunotherapies has also reported important CADRs similar to treatments with TKi, including with a high rate of skin rashes appearance of [2]. Consequently, a better understanding of the effects of such drugs on skin physiology is still necessary to manage such disorders for a better quality of life for the patient.

## Conclusion

We evaluated the effect of oncology therapy molecules at concentrations below the toxic level on epidermal development in vitro. These relevant concentrations allow us to demonstrate that oncology treatment impairs keratinocyte growth and consequently affects skin barrier. These results underlie the need of prophylaxis to support the skin barrier function during oncology therapy and consequently decrease the appearance of such CADRs.

## Data Availability

The datasets generated and/or analysed during the current study are not publicly available due Company policy but are available from the corresponding author on reasonable request.

## Abbreviations

CADR: Cutaneous adverse drug reactions
μM: Micro Molar
3D: 3-Dimensional
Cmax: Concentration maximum
DMSO: Dimethylsulfoxyde
DSG1: Desmoglein
EGFRi: Epidermal Growth Factor Receptor inhibitors
FLG: Filaggrin
HER2: Human EGF Receptor 2
IVL: Involucrin
Kd: Dissociation constant
MW: Molecular Weight
nM: Nano Molar
NSCLC: Non-small-cell lung carcinoma
PBS: Phosphate-buffered saline
PDGF: Platelet-Derived Growth Factor
RHE: Reconstructed Human Epidermis
SD: Standard deviation
SDS: Sodium Dodecyl Sulfate
SDS: Standard deviation
TEWL: Transepidermal water loss
Tki: Tyrosine kinase inhibitors
VEGFRi: Vascular Endothelial Growth Factor Receptor inhibitors
WST-8: Water-Soluble Tetrazolium Salts

## References

[1] Peus D, Hamacher L, Pittelkow MR (1997). EGF-receptor tyrosine kinase inhibition induces keratinocyte growth arrest and terminal differentiation. J Invest Dermatol..

[2] Tischer B, Huber R, Kraemer M, Lacouture ME (2017). Dermatologic events from EGFR inhibitors: the issue of the missing patient voice. Support Care Cancer..

[3] Kwak EL, Shapiro GI, Cohen SM, Becerra CR, Lenz H-J, Cheng W-F (2013). Phase 2 trial of afatinib, an ErbB family blocker, in solid tumors genetically screened for target activation. Cancer..

[4] Hanahan D, Weinberg RA (2011). Hallmarks of cancer: the next generation. Cell..

[5] Lacouture ME (2006). Mechanisms of cutaneous toxicities to EGFR inhibitors. Nat Rev Cancer..

[6] Gao X, Le X, Costa DB (2016). The safety and efficacy of osimertinib for the treatment of EGFR T790M mutation positive non-small-cell lung cancer. Expert Rev Anticancer Ther..

[7] Takeda M, Nakagawa K (2019). First-and second-generation EGFR-TKIs are all replaced to osimertinib in chemo-naive EGFR mutation-positive non-small cell lung cancer. Int J Mol Sci..

[8] Goss G, Tsai C-M, Shepherd FA, Bazhenova L, Lee JS, Chang G-C (2016). Osimertinib for pretreated EGFR Thr790Met-positive advanced non-small-cell lung cancer (AURA2): a multicentre, open-label, single-arm, phase 2 study. Lancet Oncol..

[9] Kari C, Chan TO, Rocha de Quadros M, Rodeck U (2003). Targeting the epidermal growth factor receptor in cancer: apoptosis takes center stage. Cancer Res..

[10] Mascia F, Mariani V, Girolomoni G, Pastore S (2003). Blockade of the EGF receptor induces a deranged chemokine expression in keratinocytes leading to enhanced skin inflammation. Am J Pathol..

[11] Cubero DIG, Abdalla BMZ, Schoueri J, Lopes FI, Turke KC, Guzman J (2018). Cutaneous side effects of molecularly targeted therapies for the treatment of solid tumors. Drugs Context..

[12] Peréz-Soler R, Saltz L (2005). Cutaneous adverse effects with HER1/EGFR-targeted agents: is there a silver lining?. J Clin Oncol..

[13] Yerushalmi R, Woods R, Ravdin PM, Hayes MM, Gelmon KA (2010). Ki67 in breast cancer: prognostic and predictive potential. Lancet Oncol..

[14] Sandilands A, Sutherland C, Irvine AD, McLean WHI (2009). Filaggrin in the frontline: role in skin barrier function and disease. J Cell Sci..

[15] Getsios S, Amargo EV, Dusek RL, Ishii K, Sheu L, Godsel LM (2004). Coordinated expression of desmoglein 1 and desmocollin 1 regulates intercellular adhesion. Differentiation..

[16] Steinert PM, Marekov LN (1997). Direct evidence that involucrin is a major early isopeptide cross-linked component of the keratinocyte cornified cell envelope. J Biol Chem..

[17] Paul T, Schumann C, Rüdiger S, Boeck S, Heinemann V, Kächele V (2014). Cytokine regulation by epidermal growth factor receptor inhibitors and epidermal growth factor receptor inhibitor associated skin toxicity in cancer patients. Eur J Cancer..

[18] Danilenko DM, Phillips GDL, Diaz D (2016). *In Vitro* skin models and their predictability in defining Normal and disease biology, pharmacology, and toxicity. Toxicol Pathol..

[19] Rais R, Zhao M, He P, Xu L, Deeken JF, Rudek MA (2012). Quantitation of unbound sunitinib and its metabolite N-desethyl sunitinib (SU12662) in human plasma by equilibrium dialysis and liquid chromatography–tandem mass spectrometry (LC/MS/MS): application to a pharmacokinetic study. Biomed Chromatogr..

[20] Fabian MA, Biggs WH, Treiber DK, Atteridge CE, Azimioara MD, Benedetti MG (2005). A small molecule-kinase interaction map for clinical kinase inhibitors. Nat Biotechnol..

[21] Villarroel MC, Pratz KW, Xu L, Wright JJ, Smith BD, Rudek MA (2012). Plasma protein binding of sorafenib, a multi kinase inhibitor: in vitro and in cancer patients. Invest New Drugs..

[22] Davis MI, Hunt JP, Herrgard S, Ciceri P, Wodicka LM, Pallares G (2011). Comprehensive analysis of kinase inhibitor selectivity. Nat Biotechnol..

[23] Swaisland HC, Smith RP, Laight A, Kerr DJ, Ranson M, Wilder-Smith CH (2005). Single-dose clinical pharmacokinetic studies of gefitinib. Clin Pharmacokinet..

[24] Klaeger S, Heinzlmeir S, Wilhelm M, Polzer H, Vick B, Koenig P-A, et al. The target landscape of clinical kinase drugs. Science. 2017;358 10.1126/science.aan4368. 10.1126/science.aan4368 PMC6542668 29191878

[25] Li J, Brahmer J, Messersmith W, Hidalgo M, Baker SD (2006). Binding of gefitinib, an inhibitor of epidermal growth factor receptor-tyrosine kinase, to plasma proteins and blood cells: in vitro and in cancer patients. Invest New Drugs..

[26] Togashi Y, Masago K, Fukudo M, Terada T, Ikemi Y, Kim YH (2010). Pharmacokinetics of erlotinib and its active metabolite OSI-420 in patients with non-small cell lung cancer and chronic renal failure who are undergoing hemodialysis. J Thorac Oncol..

[27] Wind S, Schnell D, Ebner T, Freiwald M, Stopfer P (2017). Clinical pharmacokinetics and pharmacodynamics of Afatinib. Clin Pharmacokinet..

[28] Hudachek SF, Gustafson DL (2013). Physiologically based pharmacokinetic model of lapatinib developed in mice and scaled to humans. J Pharmacokinet Pharmacodyn..

[29] Giri N, Masters JC, Plotka A, Liang Y, Boutros T, Pardo P (2015). Investigation of the impact of hepatic impairment on the pharmacokinetics of dacomitinib. Invest New Drugs..

[30] Reddy VP, Walker M, Sharma P, Ballard P, Vishwanathan K (2018). Development, verification, and prediction of osimertinib drug-drug interactions using PBPK modeling approach to inform drug label. CPT Pharmacometrics Syst Pharmacol..

[31] Nicholson DW, Ali A, Thornberry NA, Vaillancourt JP, Ding CK, Gallant M (1995). Identification and inhibition of the ICE/CED-3 protease necessary for mammalian apoptosis. Nature..

[32] Rosell R, Carcereny E, Gervais R, Vergnenegre A, Massuti B, Felip E (2012). Erlotinib versus standard chemotherapy as first-line treatment for European patients with advanced EGFR mutation-positive non-small-cell lung cancer (EURTAC): a multicentre, open-label, randomised phase 3 trial. Lancet Oncol..

[33] Van Schil PE, Hellmann MD, Peters S, Guidelines E (2019). ESMO Clinical Practice Guidelines for mNSCLC. Ann Oncol.

[34] Yang Y, Zhang Y, Cao Z, Ji H, Yang X, Iwamoto H (2013). Anti-VEGF- and anti-VEGF receptor-induced vascular alteration in mouse healthy tissues. Proc Natl Acad Sci U S A..

[35] Soria JC, Ohe Y, Vansteenkiste J, Reungwetwattana T, Chewaskulyong B, Lee KH (2018). Osimertinib in untreated EGFR-mutated advanced non-small-cell lung cancer. N Engl J Med..

[36] Cross DAEE, Ashton SE, Ghiorghiu S, Eberlein C, Nebhan CA, Spitzler PJ (2014). AZD9291, an irreversible EGFR TKI, overcomes T790M-mediated resistance to EGFR inhibitors in lung cancer. Cancer Discov..

[37] Chu C, Choi J, Eaby-Sandy B, Langer CJ, Lacouture ME (2018). Osimertinib: a novel dermatologic adverse event profile in patients with lung Cancer. Oncologist..

